# Characteristics of Slovenian Adults in Community-Based Whole-Food Plant-Based Lifestyle Program

**DOI:** 10.1155/2020/6950530

**Published:** 2020-07-29

**Authors:** Boštjan Jakše, Barbara Jakše, Stanislav Pinter, Jernej Pajek, Nataša Fidler Mis

**Affiliations:** ^1^Department of Food Science, Biotechnical Faculty, University of Ljubljana, Ljubljana, Slovenia; ^2^Sole Proprietor, 1230 Domžale, Slovenia; ^3^Basics of Movements in Sport, Faculty of Sport, University of Ljubljana, Ljubljana, Slovenia; ^4^Department of Nephrology, University Medical Centre Ljubljana, Ljubljana, Slovenia; ^5^Faculty of Medicine, University of Ljubljana, Ljubljana, Slovenia; ^6^Department of Gastroenterology, Hepatology and Nutrition, University Children's Hospital, University Medical Centre Ljubljana, Ljubljana, Slovenia

## Abstract

**Objective:**

Adopting a plant-based diet (PBD) and lifestyle is healthy, sustainable, and increasingly popular, while also demanding. Individuals might face challenges to maintain this lifestyle. We aimed to determine the anthropometric values and lifestyle factors and motives of adults to adopt a whole-food, plant-based (WFPB) lifestyle by joining our ongoing, community-based, WFPB lifestyle program 0.5–10 years ago.

**Methods:**

We measured body mass index (BMI) and body fat percentage status (BF%) using bioimpedance. Lifestyle status was obtained by standardized electronic questionnaires. For evaluating the motives for following strict PBD, the participants were asked to rank 8 different motives (i.e., 8: the most-, 1: the least important). *Setting*. A cross-sectional study in Slovenia. *Participants.* A total of 151 healthy adults with an average age of 39.6 years (SD: 12.5 years).

**Results:**

The participants had an average BMI of 23.9 kg/m^2^ (SD: 3.8 kg/m^2^) and an average BF% of 22.3% (SD: 7.3%), were physically very active, with an average Long International Physical Activity Questionnaire (L-IPAQ) score of 5541.2 metabolic equivalents (METs) min/week (SD: 4677.0 METs min/week), having good sleep quality, with an average Pittsburgh Sleep Quality Index (PSQI) score of 2.7 (SD: 1.8), perceiving low stress, and with an average Perceived Stress Questionnaire (PSQ) score of 0.29 (SD: 0.1). We discovered no significant differences in lifestyle between participants who were involved in our WFPB lifestyle program for short, medium, or long periods of time. The motives for WFPB lifestyle included health benefits (score: 7.9/8), body mass management (6.3), eating to satiety (4.9), convenience (4.3), environmental concerns (4.1), affordability (3.7), animal ethics (3.6), and religious reasons (1.1).

**Conclusion:**

A WFPB lifestyle program for any length of time that includes an extensive support system provides favorable, long-term lifestyle changes.

## 1. Introduction

In recent years, the adoption of a strict plant-based (vegan) diet (PBD) that is appropriately planned and supervised has become an increasing interest. New national data in Slovenia report that 1% of older adults, 3.3% of adults, and 3.1% of adolescents currently practice vegetarian diets, while only 0.4% of adolescents and adults reported practicing a strict PBD [1]. Numerous professional organizations state that an appropriately planned vegetarian diet, including a vegan diet (strict PBD), is appropriate for all life stages, including pregnancy, lactation, infancy, childhood, adolescence, and older adulthood [2–6], as well as for athletes [2, 4].

From prospective cohort studies, it was found that PB dieters had healthier lifestyle habits than nonvegetarians [7–9], and the health effects of the PBD were assumed to be strongly associated with the accompanying healthier lifestyle habits [10–19]. Many previous studies exploring the impact of long-term, strict PBDs and the associated lifestyle factors on health frequently reported healthier lifestyle behaviors [8, 20–22].

Adopting a strict PBD is demanding, with many individuals regularly facing problems and obstacles. The transition to a strict PBD may be more successful when participants are involved in a lifestyle-optimizing program providing a rationale for the diet change through lectures, prescribed meal plans, counseling, and social support [23–27].

To become long-term, motivated, and healthy plant-based dieters, individuals need to change their lifestyle behaviors. A healthy and active lifestyle is associated with health and quality of life [28], while an unhealthy lifestyle, which also includes an unhealthy diet, is recognized as a major risk factor for the development of various chronic diseases and premature mortality [29–31]. Physical activity (PA) [32], sleep hygiene [33], and stress status [34] are well-established modifiers that play key roles in health and disease prevention. These healthy lifestyle factors, among many others (e.g., moving naturally, having a purpose, belonging to a community, being in social circles that support healthy behaviors, and investing in family happiness and care), were presented as keys for longevity in studies of centenarians across diverse geographical locations (“Blue Zone” areas) [35]. A healthy lifestyle includes healthy nutrition, maintaining normal body mass (BM), avoiding smoking, limiting alcohol consumption, and being physically active; together, these factors might account for up to 50%–60% reduced risk of all-cause mortality [36]. Exercise (PA) has an important role in BM and body composition management, particularly when combined with dietary changes [37–41], and also has an independent positive impact on other aspects of health [42].

We developed a whole-food, plant-based (WFPB) lifestyle program (described in the Methods section) based on a WFPB diet and reported its effects on body composition and cardiovascular (CV) risk factors over 10–36 weeks [40, 43]. This study was part of a larger cross-sectional study looking at the diets, lifestyles, and CV risk factors of healthy participants from Slovenia who previously engaged in a Western-type diet but had been involved in our ongoing, community-based, WFPB lifestyle program for 0.5–10 years [27]. In this study, we investigated body mass index (BMI) and body fat percentage (BF%), lifestyle factors, and motives to adopt a PBD. We aimed to determine any potential differences between the participants' current BMI and BF% values, lifestyle factors, and motives for adopting their PBDs according to their length of engagement time in our program, i.e., short-term (0.5 ≤ 2 years), medium-term (2 ≤ 5 years), or long-term (5 ≤ 10 years).

## 2. Materials and Methods

### 2.1. Study Design and Eligibility

We included participants from different parts of Slovenia who voluntarily joined our ongoing, community-based, WFPB lifestyle program (see description below) 0.5–10 years ago. The study protocol was approved by the National Medical Ethics Committee (No. 0120-380/2019/17) and the Ethical Committee in the field of sports (No. 05:2019). The trial was registered at https://clinicaltrials.gov with number NCT03976479. Each participant signed an informed consent form for inclusion in the study. Participants were not remunerated financially. The study was being conducted for three months during the summer of 2019. The clinical data were gathered within two weeks.

### 2.2. Subjects

**Figure 1:**
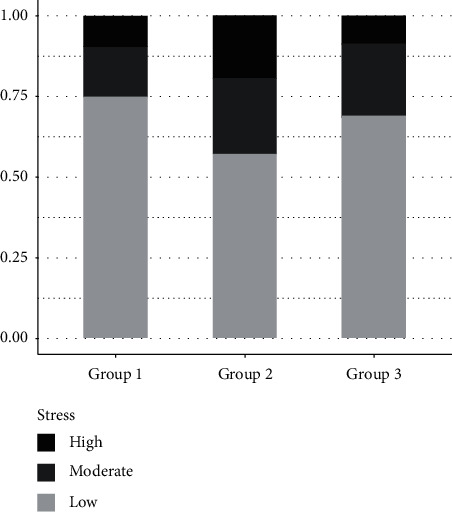
Perceived stress of all participants and according to their length of engagement time in our WFPB program.

We described subject selection in detail in our previous study which is a part of the same larger study (see text and Figure 1 on pages 3 and 4 of [27]). In short, we invited subjects (*n* = 2555) aged 18–80 years who voluntarily joined our ongoing, community-based, WFPB lifestyle program 0.5–10 years prior to study enrollment via our closed social–media support groups and by personal contact with PBD health coaches. In the present study, we included only those participants who were on a supplemented WFPB diet with ≤3% of energy from animal protein.

In this primary prevention setting, we excluded current pregnancy or lactation, current competitive or top-level athletes, major musculoskeletal restrictions, active malignant diseases (e.g., cancer, cardiovascular diseases (CVD), type 2 diabetes, autoimmune diseases, and neurodegenerative diseases), the current use of drugs for measured blood markers (e.g., plasma lipids, blood pressure, blood sugar control), >3% of energy intake from animal protein, incomplete blood assay, and unanswered questionnaires. A total of 370 participants met the inclusion criteria; 204 declined to participate.

In the final analysis, we included 151 adult participants (91% of those initially included), who were all from the same ethnic group (Caucasian race). Based on the duration of their voluntary participation in our WFPB lifestyle program, we divided participants into 3 groups: short-term (0.5–<2 years), medium-term (2–<5 years), and long-term (5–10 years).

### 2.3. WFPB Lifestyle Program

#### 2.3.1. Nutrition

The diet consisted of ≥90% of energy intake from WFPB diet and ≤10% of energy intake from plant-based meal replacement (MR) (35–37 g plant protein (soy or pea)/100 g; 1-2 portions/day) and dietary supplements (e.g., vitamin B_12_ for all participants, vitamin D_3_ in winter months, optional eicosapentaenoic acid, and docosahexaenoic acid (i.e., omega-3 long-chain polyunsaturated fatty acids). The WFPB diet was based predominately on whole or minimally processed plant foods as defined by Campbell and Campbell in 2005 [44].

#### 2.3.2. Physical Activity

It was divided into habitual, organized, and nonorganized PA. During the introduction phase, participants engaged in at least two 45-minute, guided, moderate-intensity exercise sessions weekly. After the introduction phase, participants performed the prescribed resistance workout activities by themselves. They were also encouraged to perform ≥30 minutes/day of low–moderate-intensity aerobic activity and a longer low–moderate-intensity activity during the weekend (>45 minutes, preferably 60–120 minutes), like brisk walking or hiking.

#### 2.3.3. Support System

It consisted of regular follow-ups and body composition measurements, meal plan evaluations, grocery tours, cooking workshops, assistance in the introduction of PA, individual and group support, and social media support, including (1) cooking recipes, (2) professional summaries of health and nutrition topics written in lay language, (3) posting organized group workouts and results/testimonials, and (4) a discussion board. The goal was to motivate and teach participants to improve their diet, lifestyle (i.e., quit smoking and increase PA), and consequently their well-being and health.

### 2.4. Outcomes

#### 2.4.1. Sociodemographic and Economic Status

We adopted the questionnaire provided by the National Institute of Public Health [45].

#### 2.4.2. BMI and BF% according to Time Spent in the WFPB Lifestyle Program

Participants were measured without shoes, socks, outer clothing, mobile devices, and/or keys in pockets by the same two researchers. Height (cm) was measured using the body height gauge (Kern MPE 250K100HM, Kern and Sohn, Balingen, Germany). Body composition was assessed using an 8-electrode, medically approved, calibrated, bioelectrical impedance body composition monitor (Tanita 780 S MA, Tanita Corporation, Tokyo, Japan), which provided an accurate tool to measure total BF% and fat-free mass in healthy young males and females, regardless of their level of habitual PA [46]. BMI (kg/m^2^) was also calculated. Body composition indices included BMI and BF%. According to the manufacturer's recommendations [47], before the bioimpedance measurement, participants were asked not to eat or drink for at least one hour, not to exercise for at least 24 h, or urinate for at least 30 minutes. Females were not measured three days before or after menstruation. All measurements were carried out by the same two researchers (Boštjan Jakše and Stanislav Pinter).

#### 2.4.3. Everyday Sitting, Transport Time, and PA

To assess PA, inactivity, and time spent using passive transport during the previous 7 days, we used the self-administered Long International Physical Activity Questionnaire (L-IPAQ) [48]. All study participants were instructed to maintain their preexisting PA. We emphasized the following components of the L-IPAQ: (1) time (in minutes) spent traveling in motorized transport (e.g., car, bus, and train); (2) time (in hours) spent sitting during the weekdays; (3) time (in hours) spent sitting during weekend days; (4) average daily time (in minutes, equivalent to 3.3 metabolic equivalents (METs)) performing low-intensity PA (i.e., equivalent to walking); (5) average daily time (in minutes, equivalent to 4 METs) of moderate-intensity PA; (6) average daily time (in minutes, equivalent to 8 METs) of high or vigorous-intensity PA; (7) total L-IPAQ score [49]. Areas 4–6 were related to transportation, housework/gardening, recreation, sport, and leisure PA.

The volume of activity was computed by weighting each type of activity by its energy requirements defined in metabolic equivalent of task (METs), where 1 MET is the resting metabolic rate obtained during quiet sitting, equivalent to the energy consumption of 1 kcal/kg/hour) [50]. METs are multiples of the resting metabolic rate to yield a score in MET minutes. The total number of MET minutes of PA per week is calculated as a sum of the MET minutes achieved in each category (walking-equivalent, moderate-intensity PA, and vigorous-intensity PA). For example, the MET minutes of brisk walking at 4.8 km/h PA/week would be calculated with the following formula: 3.3 METs × walking (minutes)/day × walking (days)/week) [49].

Vigorous-intensity PA is defined as achieving a minimum total PA of at least 1500 MET minutes/week, at least three days per week, or seven days per week of any combination of walking-equivalent, moderate-intensity PA, or vigorous-intensity PA to achieve a minimum total PA of at least 3000 MET minutes/week [51]. Moderate-intensity PA is defined as five or more days per week of any combination of walking-equivalent, moderate-intensity PA, or vigorous-intensity PA to achieve a minimum total PA of at least 500 MET minutes/week [52]. These amounts of vigorous-intensity PA or moderate-intensity PA equal the PA guidelines stating that at least 75 or 150 minutes/week of vigorous-intensity PA or moderate-intensity PA, respectively, should be achieved [52, 53]. A participant who did not meet the vigorous- or moderate-intensity PA level was characterized as having a “low PA” [51]. The L-IPAQ method produced a correlation of 0.8 in an assessment of test–retest repeatability [54]. We added a question regarding the weekly frequency of at least 30 minutes of organized resistance exercise to further distinguish the PA lifestyle patterns according to the general PA recommendations [53].

#### 2.4.4. Sleep Quality and Patterns

We used 19 self-rated questions from the Pittsburgh Sleep Quality Index (PSQI) questionnaire [55]. Overall, we found 9 components of the PSQI to be important for our study, while the scores of 7 components as per the scoring rules were calculated as a total score (i.e., global sleep quality), namely, (1) subjective sleep quality, (2) sleep latency, (3) sleep duration, (4) sleep efficiency, (5) sleep disturbance, (6) use of sleep medications, and (7) daytime dysfunction. The total score range was from 0 to 21, where a higher score indicated worse sleep quality and ≥5 indicated poor sleep quality [55].

#### 2.4.5. Stress Status

To measure stress status during the previous month, we used a 30-question Perceived Stress Questionnaire (PSQ) [56]. The PSQ is a valid instrument for recording subjectively perceived stress in the context of a transactional view of stress [57] and stress-related diseases [58]. The PSQ emphasizes a cognitive level of experience more than emotional states or specific life events. The questionnaire was answered using a 4-point Likert-type rating scale (1: almost never; 2: sometimes; 3: often; 4: usually). Factor analysis revealed that 7 PSQ factors defined perceived stress, namely, harassment, overload, irritability, lack of joy, fatigue, worry, and tension. Both positively and negatively formulated items were included in order to reduce acquiescent bias. After several positive items (1, 7, 10, 13, 17, 21, 25, and 29) that indicated a lack of stress, the scores were calculated in reverse and then totaled, resulting in a total raw score in the range of 30–120. A PSQ index varying from 0 (the lowest level of perceived stress) to 1 (the highest level of perceived stress) was derived from the total raw score using the following formula: PSQ index = total raw score −30/90 [56, 59]. The cutoff values for levels of stress were <0.34 for a low stress, 0.34–0.46 for moderate stress, and >0.46 for high stress [60].

#### 2.4.6. Motives for Adopting PBD

We asked participants to rank 8 different motives, where 1 was the least important and 8 was the most important: (1) health, (2) BM management/appearance, (3) environmental concerns, (4) religious reasons, (5) affordable dieting, (6) convenient dieting, (7) animal ethics, and (8) satiety/no hunger. For each motive, we calculated the average scores from the whole sample or from individual groups according to the time spent in our program.

### 2.5. Statistical Analysis

Statistical analysis was performed using R 3.5.2 with the dplyr [61], ggplot2 [62], and arsenal [63] packages. Dplyr was used for data transformation, ggplot2 for data visualization, and arsenal for statistical calculations. ANOVA was used for numerical variables with differences between groups. Tukey's post hoc test was used when the differences were statistically significant. The *t*-test was used for dependent sample analysis. For categorical variables where expected frequency in a cell was less than 5, we used Fisher's exact test; otherwise, the Chi-square test was applied. The threshold for statistical significance was less than 0.05.

## 3. Results

### 3.1. Participants' Characteristics

We included 151 adults from six regions of Slovenia and allocated them to three groups according to the duration in our WFPB lifestyle program: (1) short-term (0.5–<2 years), (2) medium-term (2–<5 years), and (3) long-term (5–10 years). The average duration spent in the WFPB lifestyle program for all participants was 4.1 years: 1.3 years for group 1, 3.9 years for group 2, and 7 years for group 3. Group 1 included 51 participants (35 females (69%) and 16 males (31%)), group 2 included 56 participants (43 females (77%) and 13 males (23%)), and group 3 included 44 participants (31 females (70%) and 13 males (30%) (*p*=0.613)). The mean ages of groups 1, 2, and 3 were 37 (SD: 14), 41 (SD: 11), and 40 (SD: 13) years, respectively.

All participants were previously engaged in a Western-type diet and lifestyle, which we assessed from a questionnaire. Most lived in a marriage or partnership status (74.9%), lived outside the Capitol (62.3%), were employed or self-employed (79.4%), and had at least mildly above-average economic status (94.7%). The majority were primary, high-school, and college-educated (48.4%). Only 2% (3) of the participants consumed alcohol (<0.5 g alcohol/day, calculated from the three-day weighted dietary protocol [27]). Regarding smoking status, 77.5% (117) of participants reported never smoking, 17.2% (26) reported still smoking, although most (20/26) reported reducing their cigarette consumption, while 4.6% stopped smoking (7/26) after adopting the WFPB lifestyle.

There were no significant differences between short-, medium-, and long-term WFPB lifestyle program participants in demographics and other characteristics, except for BF%, employment, and income status. Group 3, the long-term group, had the lowest average BF%, more self-employed participants, fewer high-school or college-educated participants, and fewer middle financial class (income from 701 to 1900 euros/month) participants (Table 1S).

### 3.2. BMI and BF% Status according to Time Spent in the WFPB Lifestyle Program

The mean BMI values of groups 1, 2, and 3 were 24.3 (SD: 4.0) kg/m^2^, 24.4 (SD: 4.0), and 22.9 (SD: 3.0) kg/m^2^ (*p*=0.103), respectively. The mean BF% values for groups 1, 2, and 3 were 22.1%, 24.4%, and 19.9% (*p*=0.009), respectively. The BMI and BF% obesity classifications are shown in Table 1. Of the group 1, 2, and 3 participants, 74.5%, 55.4%, and 79.5%, respectively, had normal BMI values [64]. A total of 69% (104) of all participants had normal BMI values, with significantly more females than males (74.3% versus 54.8% respectively, *p*=0.039). Groups 1, 2, and 3 included 7.8%, 5.4%, and 2.3% of participants from either obesity class 1 or 2, respectively. Group 2 showed 1.8% of participants in obesity class 3, but neither group 1 nor 3 included any individuals in obesity class 3. Group comparisons showed no significant differences in BMI classification status (*p*=0.087).

In terms of BF%, 88.2%, 92.9%, and 95.5% of participants in groups 1, 2, and 3 were in the normal range, respectively [65]. Group 1 included 11.8% of participants who were considered obese according to the obesity classes, whereas group 2 had 7.1% obese participants and group 3 had 4.5%. Comparisons of the groups showed no significant differences (*p*=0.446). A total of 92% (139) of participants were within the normal BF% range, with no significant difference between the genders.

### 3.3. Lifestyle Factors

Everyday Sitting, Transport Time, and PA. Participants (151) reported moderate transport time (41.7 (SD: 33.4) minutes/day), relatively low weekly and weekend prolonged daily sitting (averages of 4.0 and 5.0 hours, respectively), and high amounts of PA (1488.3, 2171.4, and 1901.9 average MET minutes/week of walking-equivalent, moderate-intensity, and vigorous-intensity PA), with a total PA level of 5541.2 MET minutes/week. There were no significant differences in everyday sitting, transport time, and PA status observed between the groups (Table 2).

A total of 16.6% (25) of participants sat for more than 8 hours/day during the week, while 9.3% (14) of participants sat for more than 8 hours/day during the weekend. In terms of exercise, 87.4% (132) of participants exceeded the recommendations for the minimum amount of moderate-intensity PA (≥500 MET minutes/week) [52], i.e., equal to 150 minutes of moderate-intensity PA/week [53]; 4.0% (6) of participants did not reach 500 MET minutes/week but performed vigorous-intensity PA according to the recommended type, frequency, and duration [53]. Therefore, 91.4% (138) of the participants performed more than the minimum amount of recommended PA, with 73.5% (111) of the participants performing more than the recommended 3000 MET minutes/week from a combination of walking-equivalent, moderate-intensity PA, or vigorous-intensity PA [51]. A total of 97.3% (147) participants performed resistance training (2.7 (SD: 0.9) times/week) for at least 30 minutes at a time.

### 3.4. Sleep Quality and Patterns

All participants had good sleep quality and patterns on average (Table 2S). Most participants went to sleep after midnight (37.7%) or before 22:00 (33.8%) and woke up before 06:30 (83.4%). They generally had either very or fairly good sleep quality (143; 94.7%), fell asleep within 15 minutes (121; 80.1%), slept for over 6 hours/night (114; 75.5%), were very sleep-efficient (146; 96.7%), and experienced sleep disturbance less than once a week or never (140; 92.7%). Three participants used sleep medications during the previous month, with 93 participants (61.6%) and 50 participants (33.1%) experiencing 1-2 and 3-4 occasions of daytime dysfunction, respectively. Overall, the participants exhibited good total sleep quality scores (2.7 (SD: 1.8)). No significant differences were observed between the groups in any category or in the total sleep quality score.

### 3.5. Stress Status

The participants' total score (average PSQ = 0.29 (SD: 0.1)) indicated low average stress levels. Furthermore, 20.5% (31) of participants experienced moderate stress status (PSQ score: 0.34–0.46), while 13.2% (20) of participants experienced severe stress status (PSQ score: >0.46). No significant differences were observed in the group comparison (Table 3S). Perceived stress levels according to the time spent in the WFPB lifestyle program are presented in Figure 1.

### 3.6. Motives for Adopting PBD

**Figure 2:**
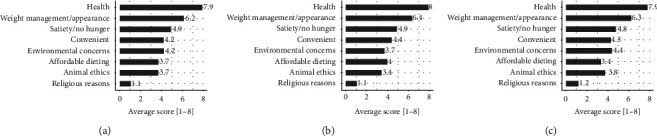
Motives for adopting PBD. (a) Group 1. (b) Group 2. (c) Group 3.

Motive scores according to the time spent in the WFPB lifestyle program are presented in Figure 2 and Table 4S. The main motive to adopt a WFPB diet was health (93.4% (141) of participants), followed by BM management/appearance and satiety/no hunger. The least important motive was religious reasons, where 90.1% (136) of participants ranked it as the least important motive for them. No significant differences in motives were observed between the groups. Group 3, including those participants who had spent the longest time in the program, showed a nonsignificant trend toward more environmental concerns and interests in animal ethics.

## 4. Discussion

### 4.1. Main Findings

We discovered no significant differences in lifestyle between the participants in our WFPB lifestyle program, regardless of the length of time they had spent in the program, potentially due to an extensive support system provided. Our results showed that it was possible to maintain a favorable balance between compliance to a supplemented WFPB diet, PA, and a healthy lifestyle. Participants implemented the proposed lifestyle changes as suggested and exhibited, on average, normal BMI and BF% values [64, 65], very limited daily sitting times, and engagement in PA above the recommended levels [51–53]. Sleep duration was slightly lower than recommended [66], but sleep quality and perceived stress levels were within recommendations [55, 60].

Only 2.0% (3) of the participants consumed alcohol (mean: <0.5 g/day) versus 70.0% of adults in the general population in Slovenia (1 g/day) [67]. In a Swiss study, alcohol abstinence was more prevalent among vegans (28%) compared to vegetarians (6.1%) or omnivores (3.2%) [68]. A total of 77.5% (117) of participants in our WFPB lifestyle program had never smoked, a much higher percentage than the general adult Slovenian population (51.3%) [67]. Furthermore, 17.2% (26) of our participants still smoked (with 76.9% smoking less and a further 21.2% having stopped completely) whereas 23.1% of adults in Slovenia were reported to still smoke [67]. A French prospective observational cohort study did not differentiate between the smoking status of vegans, vegetarians, and omnivores; however, researchers reported that there was no observed significant association between smoking status and veganism [69]. We found similar percentages of never-smokers as a Danish observational study on vegans (77.5% in our study versus 76.0%) and current smokers (17.2% in our study versus 14.7%); however, our data indicated much fewer former smokers than the Danish study (4.6% in our study versus 42.6%) [70].

Long-term beneficial lifestyle change for behavioral benefits and obesity management require an ongoing long-term intervention and support system [71] as was the case in our WFPB lifestyle program. During the past twenty years of our experiences with WFPB lifestyle program, we found the following key components for the success of our support system: (1) effective weight loss, (2) socialization, and (3) the degree of utilization of support system (i.e., nutrition, PA and received support). Firstly, a lifestyle change for behavioral benefits and obesity management require an ongoing long-term intervention and support system [71] as was already confirmed effective short- and medium-term weight loss in our previous interventional studies [40, 43]. Secondly, it was found that social integration (i.e., supportive interactions, healthy lifestyle norms) is associated with the level of involvement with the circle of influence [72]. Thirdly, there were several degrees of utilization of the support system by the participants in this study. Participants did not consume foods of animal origin (i.e., defined as ≤3% of energy from animal protein), had at least two sessions of PA weekly (i.e., moderate-intensity workout), and received support (i.e., personal consultations, body composition measurements, cooking recipes and others). We should emphasize that the shortest participation time in our program for this study was 0.5 years (i.e., the inclusion criteria) [27], as in our experience, this is the period required for participants to conquer the WFPB lifestyle.

#### 4.1.1. BMI and BF% Status

On average, our participants' BMIs were within the normal BMI range

Comparing our results with World Health Organization (WHO's) BMI [64] and BF% obesity classifications [65], fewer participants were within the normal BMI range (74.3% and 54.8% of females and males, respectively) than within the normal BF% range (92.7% and 90.5% of females and males, respectively). BMI is a poor index for the indication of total fat or fat distribution [73] and was criticized for its lack of sensitivity when distinguishing between fat mass and lean mass [74, 75]. Due to the limitations of BMI, we combined it with BF% measurements in our study, which is especially important for smaller-scale observational studies and for people with sarcopenic obesity [74, 76].

According to the BMI classification, 29.8% of our participants aged 18–78 years were overweight or obese. According to the BF% classification, only 7.9% of participants were overweight or obese. In contrast, 59% of the adult Slovenian population were overweight or obese according to BMI classifications [67].

Comparison of the participants according to the length of time in the WFPB lifestyle program (i.e., short- (0.5 ≤ 2 years), medium- (2 ≤ 5 years), or long-term (5 ≤ 10 years)) showed no significant differences in terms of BMI and %BF. However, our results are based on a cross-sectional study, while our previous nonrandomized study showed further improvement of weight loss at 9 months (i.e., 36 weeks) compared to 2.5 months (i.e., 10 weeks) [43]. A recent randomized control study (The Broad Study) using the WFPB diet in the community of obese people with ischemic heart disease or diabetes did not result in greater weight loss at 12 months compared to 6 months [77]. Several other studies argue that longer compliance to a lifestyle intervention is associated with more beneficial health outcomes [78, 79]. There are limitations for direct comparison of published results with our study due to the different nutrition (i.e., the dietary intervention in these studies was not strict PBD) and the different duration (i.e., their definition of long-term change was one year whereas ours was 5 ≤ 10 years). In addition, the improvement depends on the baseline values. Interestingly, another study used a support system and an increased intake of fruits and vegetables without limiting energy intake and evaluated the effect on the body composition of obese women. The researchers found that on average body composition did not differ between 6 and 12 months [80], despite the fact that the participants still had room for progress.

#### 4.1.2. Lifestyle Factors

Our study confirms that participants implemented a healthy lifestyle relatively quickly (i.e., already 0.5 ≤ 2 years in the program) and were able to maintain it medium- and long-term (i.e., at 2 ≤ 10 years). Centenarian and longest-lived-population studies associated a low incidence and mortality of cancer and CVD with exclusive or predominant PBD [20, 81] but also with the accompanying lifestyle behaviors [21, 35, 82]. However, it is often assumed that the health benefits of PBDs compared to omnivore diets are more related to plant-based dieters being more likely to engage in healthier lifestyle choices. Two large (the UK and the US) cross-sectional studies on plant-based dieters found that participants had lower incidences of smoking, consumed less alcohol less frequently, were more physically active, and were less sedentary [20, 21]. PB dietary intervention alone or accompanied by other lifestyle changes demonstrated impressive health benefits. For example, three randomized control trials showed major CVD (Lifestyle Heart Trial) [83, 84] and early, low-grade prostate cancer reversal in men (Prostate Cancer Lifestyle Trial) [85], where researchers predominately used a PBD as the intervention and also included large overall lifestyle changes, including PA, stress management, smoking cessation, and group psychosocial support. On the other hand, other researchers used a long-term WFPB diet as an intervention without [86] and with control groups [87], but without systematically interfering in other lifestyle components (although these factors accompany this lifestyle almost automatically); they also measured remarkable results regarding CVD reversal [86, 87].

#### 4.1.3. Everyday Sitting, Transport Time, and PA

In our study, we used a self-administered L-IPAQ questionnaire with acceptable reliability and validity when assessing the levels and patterns of PA in healthy adults in diverse settings [48, 54, 88]. We also compared both genders of older adults (over 65 years of age) [89], which was important for our sample in some cases. The L-IPAQ method produced a correlation of 0.8 in an assessment of test–retest repeatability [54].

Our results showed similar trends to the results of the Slovenian National Institute of Public Health, where sitting time was divided based on the type of work. The average weekly sitting time in our study (five and four hours/day during the week and weekend) was the same as that found for the general adult Slovenian population. In our study, 16.6% (25) and 9.3% (14) of participants sat during the week and weekend for over 8 hours/day, while the Slovenian population study reported that for simple office work and for intellectual, research, and management-type work, the average daily sitting times were 8.2 and 7.5 hours/day [67].

According to the study on the burden of diseases in 187 countries, a low level of PA was the 10th leading risk factor for morbidity [90], while daily prolonged sitting was found to have a potentially causal relationship between sedentary behavior and all-cause mortality based on several epidemiological criteria [91]. However, regular higher PA may not neutralize all of the health consequences of everyday occupational sitting [92]; in a prospective cohort study on US adults, the combination of both sitting and being less physically active was associated with up to a 94% increase in all-cause mortality in females and 48% in males compared to individuals who reported sitting for the least amount of time and being the most physically active [93].

Our participants reported moderately motorized (mostly cars) transport time, with an average of 42 minutes/day spent traveling, while 19.2% of participants reported using motorized transport <10 minutes/day.

On average, our participants exercised 5541.2 MET minutes/week. Participants in our study incorporated PA more as an integrated lifestyle component and in combination with a diet where the commitment was maintained through the assistance of an extensive support system and community network. A total of 90.7% of participants performed at least the minimum amount of weekly recommended moderate-intensity PA [52, 53], while 73% of participants performed above the recommended 3000 MET minutes/week. This is the level of PA at which the highest number of health benefits has been reported to occur [94]. While, in our study, 91.4% of participants exceeded the minimum recommendations of weekly moderate-intensity PA [52] and vigorous-intensity PA [53], data for Slovenian adults aged 25–74 years reported that only 31% and 43% of adults reached these recommended levels [67].

#### 4.1.4. Sleep Quality and Patterns

We found that the average time to go to sleep was mostly late, at 22:46, while the average waking time was 5:54 am. Participants reported falling asleep in less than 15 minutes, with the average sleep duration being fewer than seven hours per night. However, 24.5% (37) of participants reported fewer than six hours of sleep per day. In comparison, a study on the Slovenian population reported only 12.5% of adults sleeping for fewer than six hours a day [67]. Sleeping disorders represent a high-risk factor for both short- and long-term adverse health consequences in otherwise healthy individuals, as well as those with underlying diseases [95]. The dietary composition was shown to influence sleep duration, quality, and behavior [96, 97]. A recent review of epidemiological studies showed that a PBD may provide additional benefits to health via its potential effects on sleep quality but failed to establish a causal relationship between a plant-rich dietary pattern and sleep health [98]. Findings from countries across Asia, Africa [99], and Europe [100] showed that sleep disturbance was associated with socioeconomic insecurity and other environmental 24-hour lifestyle factors; thus, sleep disorders are an emerging public health issue.

#### 4.1.5. Stress Status

On average, participants in our study reported low perceived stress levels, with no significant differences observed between the three groups

Most participants (66.2%) experienced low stress, 20.5% experienced moderate stress, and 13.2% of participants reported a severe stress status. Compared to the results from the Slovenian National Institute of Public Health, our results might show a favorable stress status, particularly regarding the moderate and severe stress statuses. In comparison, bearing in mind the limitations surrounding the different methodologies, about one-quarter (23.2%) of the adult Slovenian population experienced stress daily, while 22.6% experienced severe stress challenges [67].

Stress exposure increases the risk of poor clinical outcomes across a variety of major health conditions [34]. In a recent cross-sectional survey, participants who consumed a strict PBD (vegan) reported less stress and anxiety than omnivores [7], in line with the previous cross-sectional and pilot randomized controlled studies [101, 102]. Due to different assessment methods, it was not possible to directly compare our results with these studies. However, our participants consumed high amounts of unprocessed plant foods, i.e., 1354 g/day of whole, plant-based foods [27].

#### 4.1.6. Motives for PBD

For 93% of the participants, health was the primary motive for primarily engaging in a PBD, while the least important motive was for religious reasons. Interestingly, group 3, which was the group in which participants had spent the longest amount of time on the PBD compared to groups 1 and 2, showed a nonsignificant trend toward increased motives for environmental and animal ethics reasons. These results were probably more coincidental or, according to anecdotal conversations with participants, might be based on a higher level of consciousness after achieving health or BM management goals.

According to previous studies, common motives for choosing a strict PBD (vegan) included ethical and health-related benefits, BM management, environmental concerns, and religious reasons [82, 103–105]. A study on 100 self-reported vegans living in different US states who were recruited via print and electronic advertising showed that 47% reported health benefits as their main reason for being vegan, while animal welfare, religious, and environmental reasons occurred at frequencies of 40%, 9%, and 2%, respectively [82]. In contrast, another study that recruited an international sample of 246 vegans through events and targeted social media found that ethical reasons (201 vegans) were almost fivefold more likely than health benefits (45 vegans) to be the reason to adopt a PBD [103]. A European study conducted in seven vegan supermarkets in Germany with 329 consumers following a vegan diet revealed three main motives: animal-related motives (89.7% respondents), motives related to personal well-being and/or health (69.3%), and environment-related motives (46.8%); 81.9% of respondents mentioned more than one motive. The authors concluded that dichotomously segmenting consumers into ethical versus self-oriented disregarded the fact that many consumers following a vegan diet were driven by more than one motive [104].

#### 4.1.7. Strengths and Limitations

The strengths of our study include its long-term nature, since the participants were healthy and active adults who voluntarily joined our WFPB lifestyle program 0.5–10 years ago, thereby resulting in their transformation from a Western-type, sedentary lifestyle to an active, WFPB lifestyle program. A unique feature of this study in accordance with the program is the ability to obtain detailed descriptions of their lifestyles and accompanying an extensive support system. The study also had good geographical representation, since our participants were dispersed across the country in several regions of Slovenia, including urban, suburban, and rural areas. All lifestyle measurements from the fairly large sample were performed within 14 days. Furthermore, this study was a part of an extensive project where we also investigated in detail dietary intake from foods and supplements according to nutrients and compared these data with the recommendations according to food groups. We also investigated the associations between nutrition and CV health status and some other blood markers [27]. The significance of our findings is that the presented WFPB lifestyle program has a large potential for the future from the health as well as environmental perspective. Our study advances the field of public health nutrition in terms of a clinically evaluated, long-term feasible healthy lifestyle [106] and disease prevention [13] and is fully in accordance with the proposed EAT/planetary diet, so-called “win-win” diet (i.e., win for health and win for the environment) by 2050 and beyond [107].

Our study has some obvious limitations inherent to epidemiological studies due to the possibility of under- or overreported estimates of lifestyle components assessed by questionnaires. As a limitation, there is a constant need for larger cross-sectional samples (especially males) and nonrandomized studies. In addition, we could not exclude the possible unknown impact of people within the set criteria who did not respond or were not willing to participate in the study. Additional stimuli to improve the success of the WFPB lifestyle change included the enhanced and extensive support system [27], behavior changes since the adoption of the diet [108], and motives related to personal well-being and health [109], which may all be crucial to remaining a plant-based dieter in the long-term.

## 5. Conclusions

This research was one of the first studies to systematically assess the lifestyle factors and motives in the adoption of a PBD according to the length of time that participants had spent in a community-based WFPB lifestyle program. All participants were involved in the program for 0.5–10 years and were previously on Western-type diets. On average, participants exhibited normal BMI and BF% values, were physically very active, had good sleep quality, and had low levels of perceived stress. The main motive for the WFPB dietary pattern was the expected health benefits. There were no significant differences in lifestyle status between the participants in our WFPB lifestyle program regardless of the length of time spent in the program, potentially due to the extensive support system provided.

## Figures and Tables

**Table 1:** BMI and BF% obesity classification of all participants by gender and according to their length of engagement time in our program.

	Whole sample				*p* value	Group 1		Group 2		Group 3		*p* value^‡^
Gender (*n*)	Female (109)		Male (42)		F/M							
*BMI classification* ^*∗*^	*n*	%	*n*	%	**0.039**	*n*	%	*n*	%	*n*	%	0.087
Underweight (BMI <18.5)	2	1.8	0	0		0	0	2	3.6	0	0	
Normal weight (BMI 18.5–24.9)	81	74.3	23	54.8		38	74.5	31	55.4	35	79.5	
Preobesity (BMI 25–29.9)	20	1.8	16	38.1		9	17.6	19	33.9	8	18.2	
Obesity class 1 (BMI 30–34.9)	4	3.7	2	4.8		2	3.9	3	5.4	1	2.3	
Obesity class 2 (BMI 35–39.9)	2	1.8	0	0		2	3.9	0	0	0	0	
Obesity class 3 (BMI >40)	0	0	1	2.4		0	0	1	1.8	0	0	
*BF% obesity classification* ^†^	>35%		>25%		0.739							0.446
No	101	92.7	38	90.5		45	88.2	52	92.9	42	95.5	
Yes	8	7.3	4	9.5		6	11.8	4	7.1	2	4.5	

Statistically significant values are written in bold. Fisher's exact test was used for group comparison. ^*∗*^BMI classification by the World Health Organization [64]. ^†^BF% obesity classification by the World Health Organization [65]. ^‡^Group 1 versus group 2 versus group 3 *p* values comparison.

**Table 2:** PA of all participants and according to their length of engagement time in our program.

L-IPAQ score	Whole sample		Group 1		Group 2		Group 3		*p* value
	Mean	SD	Mean	SD	Mean	SD	Mean	SD	
(1) Transportation^*∗*^ (min/day)	41.7	33.4	43.6	37.2	43.0	37.2	37.9	31.4	0.671
(2) Weekly sitting (h/day)	4.7	2.9	4.3	2.8	5.3	3.2	4.6	2.5	0.189
(3) Weekend sitting (h/day)	4.0	2.3	3.8	2.2	4.1	2.5	4.1	2.3	0.804
(4) Walking PA (MET min/week)	1488.3	1811.4	1300.5	1261.2	1544.2	2234.1	1634.9	1773.1	0.644
(5) Moderate-intensity PA (MET min/week)	2171.4	2365.0	1906.2	1712.5	1972.5	2282.6	2731.7	2996.9	0.174
(6) Vigorous-intensity PA (MET min/week)	1901.9	2819.6	2439.6	4062.7	1447.5	1839.4	1856.9	1892.1	0.191
(7) Total (MET min/week) score	5541.2	4677.0	5612.5	5181.2	4943.8	4294.4	6218.9	4534.5	0.399

Resistance workout^†^ (*n*/week)	2.7	0.9	2.8	0.9	2.5	0.8	2.8	1.0	0.084

ANOVA was used for group comparison. ^*∗*^29 (19.2%) participants used motorized transportation <10 minutes/day. ^†^An additional parameter that was not calculated to L-IPAQ.

## Data Availability

The data used to support the findings of this study are included within the article.

## Supplementary Materials

Supplementary Materials Table 1S: demographic and other characteristics of the study participants. Table 2S: sleep quality and patterns of all participants according to their length of engagement time in our program. Table 3S: perceived stress of all participants and according to their length of engagement time in our WFPB program. Table 4S: motives for adopting PBD.
