# Supplementary material for: Characteristics of Slovenian Adults in Community-Based Whole-Food Plant-Based Lifestyle Program
Source: J Nutr Metab. 2020 Jul 29;2020:6950530. doi: 10.1155/2020/6950530 (PMC7416260; doi:10.1155/2020/6950530)
Supplement: Supplementary Materials — Table 1S: demographic and other characteristics of the study participants. Table 2S: sleep quality and patterns of all participants according to their length of engagement time in our program. Table 3S: perceived stress of all participants and according to their length of engagement time in our WFPB program. Table 4S: motives for adopting PBD. [file 6950530.f1.docx]

TABLE 1S: Demographic and other characteristics of the study participants

|  | Group 1 (n = 51) | | |  | Group 2 (n = 56) | | |  | Group 3 (n = 44) | | |  | p value |
| --- | --- | --- | --- | --- | --- | --- | --- | --- | --- | --- | --- | --- | --- |
|  | *n* | | % |  | *n* | | % |  | *n* | | % |  |  |
| *Partner status* |  | | |  |  | | |  |  | | |  | 0.432 |
| Married | 18 | | 35.3 |  | 25 | | 44.6 |  | 16 | | 36.4 |  |  |
| Extramarital | 16 | | 31.4 |  | 19 | | 33.9 |  | 19 | | 43.2 |  |  |
| Single | 16 | | 31.4 |  | 12 | | 21.4 |  | 8 | | 18.2 |  |  |
| Widowed | 1 | | 2.0 |  | 0 | | 0 |  | 1 | | 2.3 |  |  |
| *Living regions* |  | | |  |  | | |  |  | | |  | - |
| Capital city (Central) | 26 | | 51.0 |  | 13 | | 23.2 |  | 18 | | 40.9 |  |  |
| Dolenjska (South East) | 12 | | 23.5 |  | 28 | | 50.0 |  | 11 | | 25.0 |  |  |
| Gorenjska (North/North West) | 4 | | 7.8 |  | 6 | | 10.7 |  | 6 | | 13.6 |  |  |
| Notranjska (Central/South West) | 1 | | 2.0 |  | 0 | | 0 |  | 2 | | 4.5 |  |  |
| Prekmurje (North East) | 1 | | 2.0 |  | 0 | | 0 |  | 0 | | 0 |  |  |
| Štajerska (East) | 7 | | 13.7 |  | 9 | | 16.1 |  | 7 | | 15.9 |  |  |
| *Living environment* |  | | |  |  | | |  |  | | |  | 0.543 |
| City | 14 | | 27.5 |  | 13 | | 23.2 |  | 14 | | 31.8 |  |  |
| Suburban | 13 | | 25.5 |  | 19 | | 33.9 |  | 16 | | 36.4 |  |  |
| Rural | 24 | | 47.1 |  | 24 | | 42.9 |  | 14 | | 31.8 |  |  |
| *Education* |  | | |  |  | | |  |  | | |  | 0.347 |
| Primary /High School | 25 | | 49.0 |  | 17 | | 30.4 |  | 15 | | 34.1 |  |  |
| College | 4 | | 7.8 |  | 9 | | 16.1 |  | 3 | | 6.8 |  |  |
| Bachelor/University degree | 20 | | 39.2 |  | 28 | | 50.0 |  | 24 | | 54.5 |  |  |
| Masters/PhD degree | 1 | | 2.0 |  | 2 | | 3.6 |  | 2 | | 4.5 |  |  |
| N/A (did not disclose) | 1 | | 2.0 |  | 0 | | 0 |  | 0 | | 0 |  |  |
| *Employment status* |  | | |  |  | | |  |  | | |  | **0.021** |
| Employed/help in family business | 30 | | 58.8 |  | 39 | | 69.6 |  | 24 | | 54.5 |  |  |
| Self-employed/contract | 5 | | 9.8 |  | 9 | | 16.1 |  | 13 | | 29.5 |  |  |
| Retired | 2 | | 3.9 |  | 3 | | 5.4 |  | 2 | | 4.5 |  |  |
| High-school/student | 12 | | 23.5 |  | 1 | | 1.8 |  | 3 | | 6.8 |  |  |
| Unemployed | 2 | | 3.09 |  | 3 | | 5.4 |  | 2 | | 4.5 |  |  |
| *Economic status* |  | | |  |  | | |  |  | | |  | 0.402 |
| Low income/financially challenged | 4 | | 7.8 |  | 3 | | 5.4 |  | 1 | | 2.3 |  |  |
| Average/mildly above average | 45 | | 88.2 |  | 50 | | 89.3 |  | 42 | | 97.7 |  |  |
| Above average | 2 | | 3.9 |  | 3 | | 5.4 |  | 1 | | 2.3 |  |  |
| *Income (€)* |  | | |  |  | | |  |  | | |  | **0.040** |
| ≤700 | 7 | | 14.0 |  | 2 | | 3.6 |  | 0 | | 0 |  |  |
| 701–1100 | 7 | | 14.0 |  | 3 | | 5.4 |  | 2 | | 6.8 |  |  |
| 1101–1500 | 6 | | 12.0 |  | 8 | | 14.3 |  | 5 | | 11.3 |  |  |
| 1501–1900 | 13 | | 26.0 |  | 12 | | 21.4 |  | 6 | | 13.6 |  |  |
| ≥ 1901 | 17 | | 34.0 |  | 31 | | 55.4 |  | 29 | | 65.9 |  |  |
| N/A (did not disclose) | 0 | | 0 |  | 0 | | 0 |  | 1 | | 2.3 |  |  |
| *Smoking* |  | | |  |  | | |  |  | | |  | 0.580 |
| Current | 7 | | 13.7 |  | 9 | | 16.1 |  | 10 | | 22.7 |  |  |
| Former | 4 | | 7.8 |  | 2 | | 3.6 |  | 1 | | 2.3 |  |  |
| Never | 40 | | 78.4 |  | 44 | | 78.5 |  | 33 | | 75.0 |  |  |
| N/A (did not disclose) | 0 | | 0 |  | 1 | | 1.8 |  | 0 | | 0 |  |  |
| *Years of smoking*^†^ |  | | |  |  | | |  |  | | |  | 0.326 |
| ≤5 | 1 | | 2.0 |  | 2 | | 3.6 |  | 1 | | 2.3 |  |  |
| 6-10 | 1 | | 2.0 |  | 3 | | 5.4 |  | 5 | | 11.4 |  |  |
| 11-20 | 5 | | 9.8 |  | 2 | | 3.6 |  | 4 | | 9.1 |  |  |
| >20 | 0 | | 0 |  | 2 | | 3.6 |  | 0 | | 0 |  |  |
| Less than before PBD | 5 | | 71.4 |  | 8 | | 88.9 |  | 7 | | 70.0 |  |  |
| *Menstrual status* |  | | |  |  | | |  |  | | |  | 0.435 |
| Menstruation | 29 | | 82.9 |  | 32 | | 74.4 |  | 23 | | 74.2 |  |  |
| Perimenopause | 2 | | 5.7 |  | 0 | | 0 |  | 2 | | 6.5 |  |  |
| Menopause | 4 | | 11.4 |  | 11 | | 25.6 |  | 6 | | 19.4 |  |  |
| *Medications* |  |  | |  |  |  | |  |  |  | |  |  |
| Thyroid medication | 1 | | 2.0 |  | 0 | | 0 |  | 1 | | 2.3 |  | - |
| Birth-control pills | 1 | | 2.0 |  | 0 | | 0 |  | 1 | | 2.3 |  | - |
| Vitamin D_3_ prescribed by doctor^††^ | 2 | | 4.0 |  | 1 | | 1.8 |  | 1 | | 2.3 |  | - |
| Nausea | 0 | | 0 |  | 0 | | 0 |  | 1 | | 2.3 |  | - |
| Others | 1 | | 2.0 |  | 0 | | 0 |  | 1 | | 2.3 |  | - |
| Total | 5 | | 10.0 |  | 1 | | 1.8 |  | 5 | | 11.3 |  | - |
| Alcohol consumption^‡^ | 2 | | 3.9 |  | 1 | | 1.8 |  | 0 | | 0 |  | - |
|  | Mean | | SD |  | Mean | | SD |  | Mean | | SD |  |  |
| Quantity (mL/d) | 0.2 | | 0.8 |  | 0 | | 0 |  | 0.7 | | 1.0 |  | 0.527 |

Statistically significant values are written bold. For categorical variables where expected frequency in a cell was less than 5, we used Fisher’s exact test, otherwise we used Chi-square test. ^†^ Only current smokers included. ^††^ Vitamin D_3_ here represents only medically prescribed vitamin D. ^‡^ Measured by three-day weighted dietary record.

TABLE 2S: Sleep quality and patterns of all participants according to their length of engagement time in our program

| PSQI score | | | | | | | | | | | | |
| --- | --- | --- | --- | --- | --- | --- | --- | --- | --- | --- | --- | --- |
|  | Whole sample | | |  | | Group 1 | | Group 2 | | Group 3 | | p value |
|  | *n* | % | |  | | *n* | % | *n* | % | *n* | % |  |
| *Time (h)* |  | |  | | Sleep time | | | | | | | 0.164 |
| 21:01–22:00 | 51 | 33.8 | |  | | 17 | 33.3 | 14 | 25.0 | 20 | 45.5 |  |
| 22:01–23:00 | 25 | 16.6 | |  | | 6 | 11.8 | 12 | 21.4 | 7 | 15.9 |  |
| 23:01–00:00 | 18 | 11.9 | |  | | 5 | 9.8 | 6 | 10.7 | 7 | 15.9 |  |
| After midnight | 57 | 37.7 | |  | | 23 | 45.1 | 24 | 42.9 | 10 | 22.7 |  |
| *Time (h)* |  | |  | | Wake-up time | | | | | | | 0.549 |
| 4:00–5:30 | 58 | 38.4 | |  | | 19 | 37.3 | 24 | 42.9 | 15 | 34.1 |  |
| 5:31–6:30 | 68 | 45.0 | |  | | 24 | 47.1 | 26 | 46.4 | 18 | 40.9 |  |
| 6:21–6:59 | 3 | 2.0 | |  | | 1 | 2.0 | 0 | 0 | 2 | 4.5 |  |
| From 7:00 | 22 | 14.6 | |  | | 7 | 13.7 | 6 | 10.7 | 9 | 20.5 |  |
| *Component 1* |  | |  | | Subjective sleep quality | | | | | | | 0.959 |
| Very and fairly good | 143 | 94.7 | |  | | 48 | 94.1 | 53 | 94.6 | 44 | 95.5 |  |
| Fairly and very bad | 8 | 5.3 | |  | | 3 | 5.9 | 3 | 5.4 | 2 | 4.5 |  |
| *Component 2* |  |  | |  | | Sleep latency | | | | | | 0.512 |
| ≤15 min | 121 | 80.1 | |  | | 38 | 74.5 | 47 | 83.9 | 36 | 81.8 |  |
| 16–30 min | 26 | 17.2 | |  | | 11 | 21.6 | 7 | 12.5 | 8 | 18.2 |  |
| 31–60 min | 4 | 2.6 | |  | | 2 | 3.9 | 2 | 3.6 | 0 | 0 |  |
| *Component 3* |  |  | |  | | Sleep duration | | | | | | 0.545 |
| More than 7 hours | 44 | 29.1 | |  | | 18 | 35.3 | 12 | 21.4 | 14 | 31.8 |  |
| 6–7 hours | 70 | 46.4 | |  | | 21 | 41.2 | 28 | 50.0 | 21 | 47.7 |  |
| Less than 6 hours | 37 | 24.5 | |  | | 12 | 23.5 | 16 | 28.6 | 9 | 20.5 |  |
| *Component 4* |  |  | |  | | Sleep efficiency | | | | | | 0.072 |
| <65% | 0 | 0 | |  | | 0 | 0 | 0 | 0 | 0 | 0 |  |
| 65%–74% | 2 | 1.3 | |  | | 0 | 0 | 1 | 1.8 | 1 | 2.3 |  |
| 75%–84% | 3 | 2.0 | |  | | 0 | 0 | 0 | 0 | 3 | 6.8 |  |
| >85% | 146 | 96.7 | |  | | 51 | 100.0 | 55 | 98.2 | 40 | 90.9 |  |
| *Component 5* |  |  | |  | | Sleep disturbance | | | | | | 0.312 |
| Not during the past month | 16 | 10.6 | |  | | 5 | 9.8 | 9 | 16.1 | 2 | 4.5 |  |
| Less than once/week | 124 | 82.1 | |  | | 41 | 80.4 | 44 | 78.6 | 39 | 88.6 |  |
| Once or twice/week | 10 | 6.6 | |  | | 5 | 9.8 | 3 | 5.4 | 2 | 4.5 |  |
| Three or more times/week | 1 | 0.7 | |  | | 0 | 0 | 0 | 0 | 1 | 2.3 |  |
| *Component 6* |  |  | |  | | Sleep medication use | | | | | | 0.554 |
| Not during the past month | 148 | 98.0 | |  | | 50 | 98.0 | 55 | 98.2 | 43 | 97.7 |  |
| Less than once/week | 1 | 0.7 | |  | | 1 | 2.0 | 0 | 0 | 0 | 0 |  |
| Three or more times/ week | 2 | 1.3 | |  | | 0 | 0 | 1 | 1.8 | 1 | 2.3 |  |
| *Component 7* |  |  | |  | | Daytime dysfunction | | | | | | 0.276 |
| 1–2 days | 93 | 61.6 | |  | | 34 | 66.7 | 33 | 58.9 | 26 | 59.1 |  |
| 3–4 days | 50 | 33.1 | |  | | 12 | 23.5 | 21 | 37.5 | 17 | 38.6 |  |
| 5–6 days | 5 | 5.3 | |  | | 4 | 7.8 | 1 | 1.8 | 0 | 0 |  |
| Every day | 3 | 2.0 | |  | | 1 | 2.0 | 1 | 1.8 | 1 | 2.3 |  |
|  | Score | SD | |  | | Score | SD | Score | SD | Score | SD |  |
| *Global sleep quality^*^* | 2.7 | 1.8 | |  | | 2.8 | 1.7 | 2.5 | 1.8 | 2.7 | 2.1 | 0.745 |

* Sum of seven components score (range: 0–21; ≥5 indicate poor sleep quality). For categorical variables where expected frequency in a cell was less than 5, we used Fisher’s exact test, otherwise we used Chi-square test.

TABLE 3S: Perceived stress of all participants and according to their length of engagement time in our WFPB program

| PSQ score | | | | | | | | | | | | | |
| --- | --- | --- | --- | --- | --- | --- | --- | --- | --- | --- | --- | --- | --- |
|  | Whole sample | |  |  | Group 1 | |  | Group 2 | |  | Group 3 | | p value |
|  | Mean | SD |  |  | Mean | SD |  | Mean | SD |  | Mean | SD |  |
| Positive items score | 17.3 | 3.9 |  |  | 17.0 | 3.9 |  | 17.9 | 4.2 |  | 16.8 | 3.8 | 0.330 |
| Total PSQ index score^*^ | 0.29 | 0.1 |  |  | 0.27 | 0.1 |  | 0.32 | 0.1 |  | 0.28 | 0.1 | 0.077 |
|  | *n* | % |  |  | *n* | % |  | *n* | % |  | *n* | % |  |
| Stress status |  |  |  |  |  |  |  |  |  |  |  |  | 0.298 |
| Low | 100 | 66.2 |  |  | 38 | 74.5 |  | 32 | 57.1 |  | 30 | 68.2 |  |
| Moderate | 31 | 20.5 |  |  | 8 | 15.7 |  | 13 | 23.2 |  | 10 | 22.7 |  |
| Severe | 20 | 13.2 |  |  | 5 | 9.8 |  | 11 | 19.6 |  | 4 | 9.1 |  |

^*^ Stress levels: <0.34 (low stress), 0.34–0.46 (moderate stress), and >0.46 (high stress). ANOVA was used for Positive items score and Total PSQ index score for groups comparison. Fisher’s exact test was used for Stress status for groups comparison.

TABLE 4S: Motives for adopting PBD

|  | Whole sample | |  | Group 1 | |  | Group 2 | |  | Group 3 | | p value |
| --- | --- | --- | --- | --- | --- | --- | --- | --- | --- | --- | --- | --- |
|  | Score (1–8)^*^ | | | | | | | | | | |  |
|  | Mean | SD |  | Mean | SD |  | Mean | SD |  | Mean | SD |  |
| Health | 7.9 | 0.3 |  | 7.9 | 0.3 |  | 8.0 | 0.2 |  | 7.9 | 0.4 | 0.290 |
| BM management/Appearance | 6.3 | 1.5 |  | 6.2 | 1.5 |  | 6.4 | 1.2 |  | 6.3 | 1.5 | 0.691 |
| Satiety/No hunger | 4.9 | 1.7 |  | 4.9 | 1.6 |  | 4.9 | 1.7 |  | 4.8 | 1.7 | 0.935 |
| Convenient dieting | 4.3 | 1.3 |  | 4.2 | 1.4 |  | 4.4 | 1.3 |  | 4.3 | 1.1 | 0.728 |
| Environmental concerns | 4.1 | 1.8 |  | 4.2 | 1.8 |  | 3.7 | 1.7 |  | 4.4 | 1.8 | 0.119 |
| Affordable dieting | 3.7 | 1.4 |  | 3.7 | 1.6 |  | 4.0 | 1.2 |  | 3.4 | 1.2 | 0.083 |
| Animal ethics | 3.6 | 1.7 |  | 3.7 | 1.5 |  | 3.4 | 1.7 |  | 3.8 | 1.8 | 0.560 |
| Religious reasons | 1.1 | 0.4 |  | 1.1 | 0.3 |  | 1.1 | 0.3 |  | 1.2 | 0.7 | 0.372 |

^*^ 1: the least, 8: the most important. ANOVA vas used for groups comparison.
